# Patterns of medical intermediate care unit use in critically ill very old patients in France

**DOI:** 10.1007/s41999-026-01495-x

**Published:** 2026-05-13

**Authors:** Adrien Migeon, Julien Demiselle, Lucile Godillon, Victoire Leroy, Leslie Grammatico-Guillon, Antoine Guillon

**Affiliations:** 1 Department of Geriatrics, Tours University Hospital, Tours, France; 2 University of Strasbourg (UNISTRA), Department of Medicine, FHUTARGET, Medical Intensive Care Unit, Strasbourg University Hospital, Hautepierre Hospital, Strasbourg, France; 3 UMR 1260, INSERM (French National Institute of Health and Medical Research), Regenerative Nanomedicine (RNM), FMTS, Strasbourg, France; 4 Clinical Data Center, Department of Public Health, Epidemiology Unit EpiDcliC, Tours University Hospital, Tours, France; 5 INSERM (French National Institute of Health and Medical Research), UMR 1253, iBrain, University of Tours, Tours, France; 6 Research Unit MAVIVH, INSERM U1259, Medical School, University of Tours, Tours, France; 7 Intensive Care Unit, Tours University Hospital, University of Tours, 2 Bd Tonnellé, 37044 Tours Cedex 9, Tours, France; 8 INSERM (French National Institute of Health and Medical Research), UMR 1100, Research Center for Respiratory Diseases (CEPR), University of Tours, Tours, France

**Keywords:** Critical care, Intensive care unit, Intermediate care units, Old patients, Long-term mortality

## Abstract

**Purpose:**

The growing population of adults aged ≥ 80 represents major challenges for critical care. Intermediate care units (IMCUs) offer a level that could bridge the gap between ICU and standard ward, but information is lacking regarding IMCU use for older critically ill patients. This study aimed to characterize critical-care trajectories involving IMCU use among older critically ill patients at a national level.

**Methods:**

A nationwide retrospective cohort study was conducted in France, including all patients hospitalized in medical critical care in 2018. Patients were categorized as young (18–64), old (65–79), and very old adults (≥ 80). Trajectories between critical care units and general wards were visualized using Sankey diagrams. Overall survival was represented with Kaplan–Meier curves according to trajectory patterns.

**Results:**

Of 439,213 individuals admitted to medical critical care, 28.2% were aged ≥ 80. Among patients aged ≥ 80 admitted to an ICU, a step-up strategy (IMCU before ICU) was used in 12% of patients, and a step-down strategy (ICU followed by IMCU) in 23%. The step-up strategy was associated with high mortality in critical care (46.6%, compared to 33.9% for patients admitted directly to an ICU). Step-down patients had an extended critical care length of stay; however, those discharged from critical care via step-down strategy showed a lower cumulative mortality at one year (RR 0.86).

**Conclusion:**

IMCUs play a central role in the management of very old critically ill patients. The potential benefit of IMCU care for older adults transitioning out of the ICU should be evaluated in prospective clinical trials.

**Supplementary Information:**

The online version contains supplementary material available at https://doi.org/10.1007/s41999-026-01495-x.

## Key summary points

**Aim:**

To characterize critical-care trajectories involving IMCU use among older critically ill patients at a national level.

**Findings:**

Step-up strategy (IMCU as an initial assessment setting followed by transfer to the ICU) was associated with higher mortality in this population. Step-down strategy (IMCU as a transitional care setting following ICU discharge) was associated with more favorable outcomes.

**Message:**

Transition patterns involving IMCUs within the critical-care pathway demonstrated contrasting associations with mortality among very old patients. A step-up strategy was associated with higher mortality, whereas a step-down strategy was associated with more favorable outcomes.

**Supplementary Information:**

The online version contains supplementary material available at https://doi.org/10.1007/s41999-026-01495-x.

## Introduction

Worldwide, the population continues to age; the number of individuals aged 80 years or older is expected to triple by 2050 [1]. In this context, care trajectories of older critically ill patients represent an increasingly important global challenge [1]. Previous research demonstrated that intensive care unit (ICU) admissions among very old patients have increased worldwide [2] and even doubled over the last decade in France [3]. Furthermore, nearly one third of patients admitted to intermediate care units (IMCU) are aged over 80 years, underscoring the expanding role of intermediate care for this specific population [4].

**Fig. 1:**
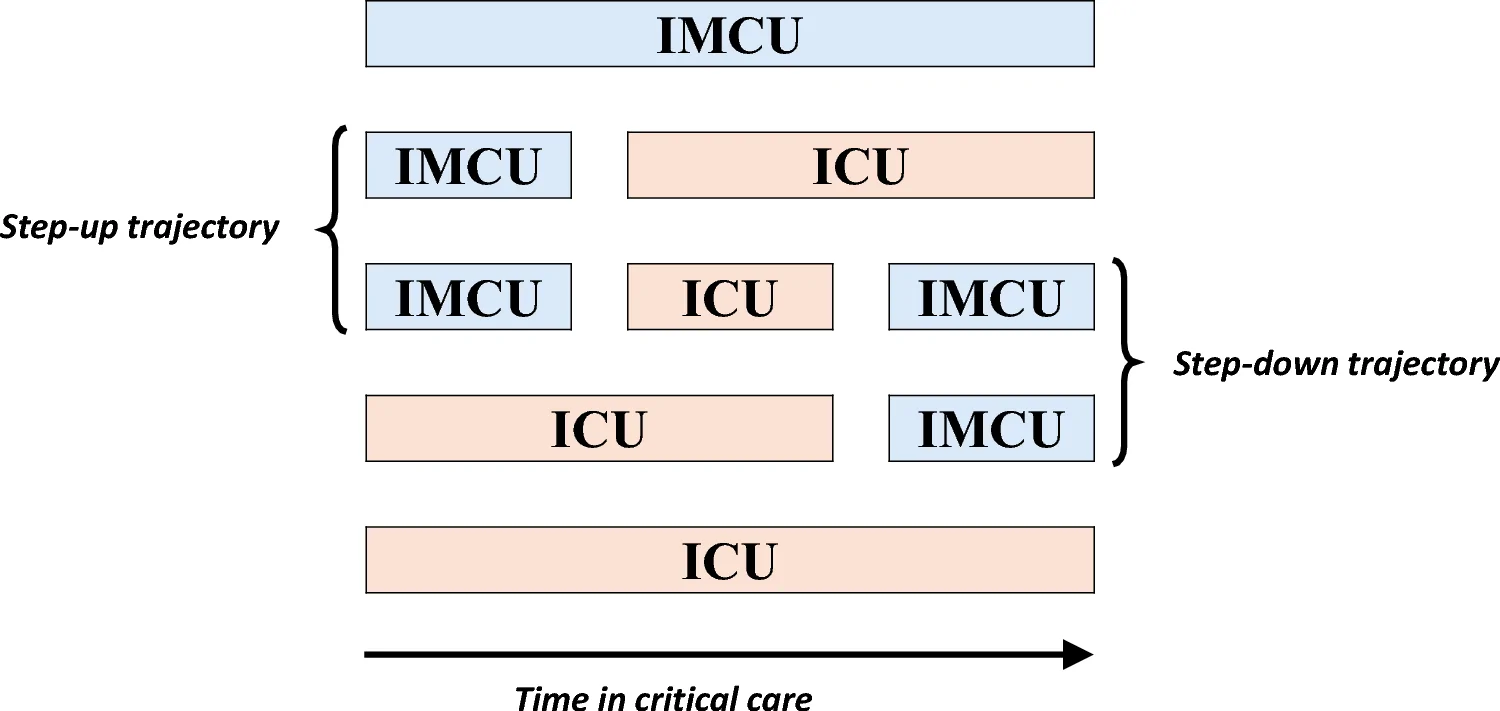
Definition of trajectories and transition patterns within the critical care system in France. *ICU* intensive care unit; *IMCU* intermediate care unit

IMCUs are designed for patients requiring close monitoring but limited organ support. They have a level of nursing staff (and costs) lower than ICU although higher than general wards and can manage patients that may require temporary life-support methods, pending if necessary a transfer to the ICU. Specific criteria have been defined for admitting patients to ICU or IMCU [5, 6]; however, determining the critical-care trajectories for a very old patient stands as one of the most intricate choices an intensivist must confront [7–11]. Patients older than 80 years old (y.o.) in ICU have a 1-year mortality rate close to 50%, and survivors have frequent functional decline [4, 12–15]. Indeed, this decision requires careful balancing between the risk of a sole age-based exclusion from ICU admission and the risk of initiating disproportionate or non-beneficial intensive treatments. Consequently, IMCU offers a level of care that could bridge the gap between the high intensity of the ICU and the standard hospital wards, and appears appropriate for the management of older critically ill patients. Three scenarios involving IMCU hospitalization can be anticipated for critically ill patients: (i) IMCU as an alternative to ICU admission when ICU care is denied; (ii) IMCU as an initial assessment setting, with subsequent transfer to ICU for patients who clinically deteriorate (step-up trajectory); and (iii) IMCU as a transitional care setting for patients discharged from ICU (step-down trajectory) (Fig. 1).

Information is lacking to clearly decipher these critical-care trajectories for older critically ill patients. In a nationwide French study including all patients aged 80 years or older hospitalized in IMCUs, we demonstrated that an important proportion of older patients met the criteria for ICU admission, despite not being transferred to the ICU [11]. Regarding the step-up strategy, occurrences from 33 to 80% are reported, suggesting that this group of patients represents a substantial proportion of admissions but no specific information is available for the older critically ill patients [16]. The step-down strategy has recently emerged as an interesting solution for old patients transitioning out of intensive care, as IMCUs are associated with fewer iatrogenic complications, less disruption of sleep and fewer invasive monitoring tools. In 2025, a hospital in France implemented a pilot “post–intensive care geriatric unit” co-managed by geriatricians and intensivists. Located close to the ICU, this unit presents a higher staff-to-patient ratio and enables continuous vital monitoring [17].

Recent European consensus-based recommendations for the management of very old patients in intensive care highlight the need to better characterize care trajectories and transition patterns within the critical care system [18]. The aim of this study was to identify and characterize critical-care trajectories involving IMCU among old critically ill patients at a national French level.

## Methods

### Design, data collection and study population

A retrospective cohort study was conducted using data from the national French Hospital Discharge Database (HDD). Access to linked de-identified data in the HDD was performed in accordance with the French Reference Methodology procedure MR-005 for retrospective HDD studies, declaration signed by the teaching hospital of Tours (MR005 number I4116221019), as regulated by the French Data Protection Board (*Commission Nationale de l’Informatique et des Libertés*, CNIL). According to French regulations, consent or information of each patient included was not required to use the French HDD de-identified data. As each patient is assigned a unique identification number, reconstruction of their care pathway over time was performed as previously described [12, 19–21]. We selected all patients aged ≥ 18 years hospitalized in Metropolitan France in critical care between January 1 and December 31, 2018. Patients in surgical critical care units and those with organ donations were not included in the study, as the prognosis for planned perioperative situations is already well-documented and differs significantly from acute organ dysfunction in very old patients [4, 22, 23].

### Variables of interest

For each inpatient stay, the following variables were collected: age, sex, pre-critical care location, hospitalization location, length of stay, unit discharge, vital status at critical care discharge and 1-year survival. Age was categorized according to WHO recommendations and previous literature: young adults (18–64 years), older adults (65–79 years), and very old adults (≥ 80 years) [13]. ICD-10 diagnosis codes (International Classification of Diseases, 10th Revision) were collected from the previous two years to calculate the Charlson Comorbidity Index (CCI) for all patients [24, 25] and frailty was estimated using the validated Hospital Frailty Risk Score (HFRS) for patients above 65 years old [10, 26, 27]. Reasons for admission to critical care were also collected using the primary ICD-10 codes and classified by organ system (Supplementary Table S1). SAPS II (Simplified Acute Physiology Score II) was extracted from the hospital records and analyzed as a categorical variable as previously proposed [10, 11, 28, 29]. High-level organ supports, including inotropic or vasopressor support and invasive or non-invasive mechanical ventilation, were identified using the French common procedure terminology codes (French CPT) [10, 11, 29] (Supplementary Table S2). Hospital stays were categorized according to the type of healthcare unit: ICU and IMCU (Supplementary Table S3), while all other conventional medical units were aggregated into a single category. Five trajectories and transition patterns within the critical care system were defined: (i) ICU alone, (ii) step-up trajectory (IMCU followed by ICU), (iii) step-down trajectory (ICU followed by IMCU), (iv) both step-up and step-down trajectory (IMCU followed by ICU followed by IMCU), and (v) IMCU alone (Fig. 1).

### Statistical analysis

Categorical variables were summarized as frequencies and percentages, while continuous variables were expressed as means (standard deviation, SD) or medians (interquartile range, IQR) based on their distribution. Pre-critical care location, the five trajectories defined, and vital status at critical care discharge were used to generate a global Sankey diagram, as well as age-stratified flow descriptions. All available data were used to describe the epidemiological characteristics of these groups according to trajectory patterns.

Statistical tests for direct comparisons within the study population were not performed because the analysis included the entire target population rather than a sample. In this context, there is no sampling variability to account for, making descriptive comparisons sufficient.

We conducted two complementary analyses to assess the association between step-up and step-down strategies and one-year mortality in very old patients. Patients with a step-up trajectory (IMCU → ICU with or without subsequent step-down) were compared with those without step-up (ICU alone or ICU → IMCU). Patients with a step-down trajectory (ICU → IMCU with or without prior step-up) were compared with those without step-down (ICU alone or IMCU → ICU). For the step-down analysis, mortality before discharge from critical care would introduce immortal time bias, as patients must survive the ICU stay to be eligible for step-down. Thus a landmark approach at critical care discharge was applied, restricting the analysis to patients alive at that time. Overall survival after critical care was estimated with Kaplan–Meier curves. Because the proportional hazards assumption was not met, Kaplan–Meier estimates were presented and Cox model–derived hazard ratios were not calculated. All analyses were performed using R (version 4.3.0).

## Results

**Fig. 2:**
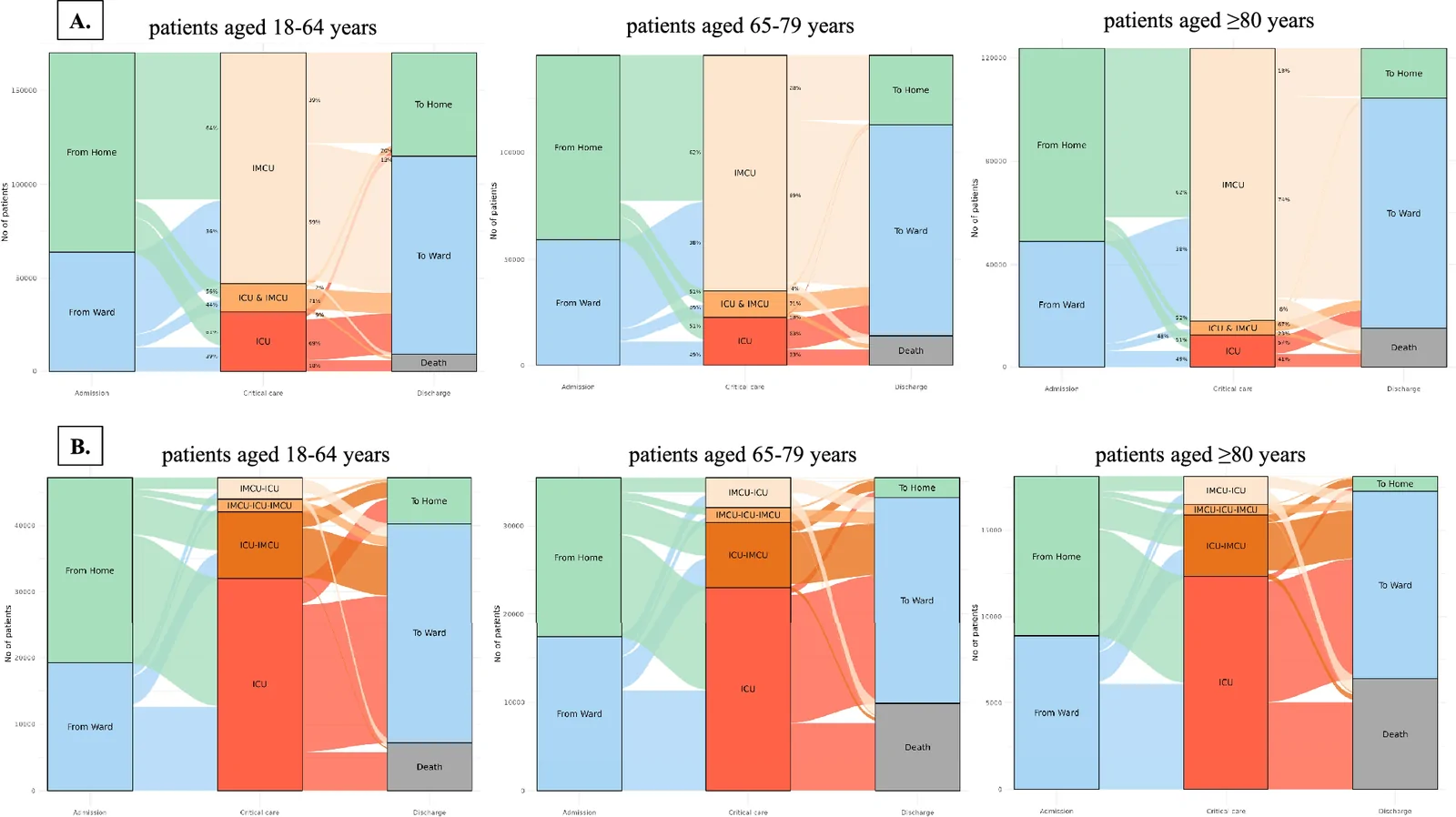
Graphical representation of trajectories between critical care units and wards according to age groups and visualized using Sankey diagrams, France. **A** Trajectory before and after critical care. **B** Trajectory before and after ICU of patients admitted at least once to the ICU, according to age groups

During the 1-year study period, 439,213 individuals were admitted to medical critical care in France; 28.2% (*n* = 123,692) were aged 80 years or older. In the overall adult population hospitalized in critical care, 15.3% (*n* = 67,359) were exclusively admitted to the ICU, 77.0% (*n* = 338,400) to the IMCU, and 7.7% (*n* = 33,454) experienced both IMCU and ICU stays (Supplementary Table S4). However, this distribution varied markedly across age classes. ICU admission was more often preferred for younger adults, whereas the oldest age class was more frequently admitted to the IMCU (Fig. 2A). IMCU utilization was 72.3% among critically ill adults aged 18–64 years, compared with 85.4% among those aged 80 years and older (Table 1; Supplementary Table S5).

**Table 1:** Characteristics of patients aged 80 years or older hospitalized in critical care units in France, 2018

	ICU *N* = 18,119 (14.6%)	IMCU *N* = 105,573 (85.4%)	All *N* = 123,692 (100%)
Men (*n*, %)	9571 (52.8%)	46,861 (44.4%)	56,432 (45.6%)
Age class (*n*, %)			
80–84 years old	9780 (54.0%)	42,323 (40.1%)	52,103 (42.1%)
85–89 years old	6290 (34.7%)	39,282 (37.2%)	45,572 (36.8%)
≥ 90 years old	2049 (11.3%)	23,968 (22.7%)	26,017 (21%)
Mean ± std	84.7 ± 3.7	86.2 ± 4.4	86 ± 4.3
Median [Q1–Q3]	84 [82–87]	86 [83–89]	85 [83–89]
Charlson Comorbidity Index (*n*, %)			
Low 0	8651 (47.7%)	56,023 (53.1%)	64,674 (52.3%)
Middle [1, 2]	4504 (24.9%)	24,601 (23.3%)	29,105 (23.5%)
High ≥ 3	4964 (27.4%)	24,949 (23.6%)	29,913 (24.2%)
Median [Q1–Q3]	2 [1–3]	2 [0–3]	2 [1–3]
Hospital Frailty Risk Score (*n*, %)			
Low < 5	11,267 (62.2%)	69,204 (65.6%)	80,471 (65.1%)
Middle [5—15[	4677 (25.8%)	24,464 (23.2%)	29,141 (23.6%)
High ≥ 15	2,175 (12.0%)	11,905 (11.3%)	14,080 (11.4%)
Median [Q1–Q3]	6.1 [2.4–12.1]	5.8 [2.1–12.1]	5.9 [2.2–12.1]
Primary diagnosis registered in critical care (n, %)			
Respiratory	6738 (37.2%)	17,543 (16.6%)	24,281 (19.6%)
Cardiovascular	4641 (25.6%)	43,251 (41.0%)	47,892 (38.7%)
Neurologic	2146 (11.8%)	23,278 (22.0%)	25,424 (20.6%)
Other	4594 (25.4%)	21,501 (20.4%)	26,095 (21.1%)
SAPS II class (*n*, %)			
< 40	5035 (27.8%)	90,661 (85.9%)	95,696 (77.4%)
[40—50[	4126 (22.8%)	9627 (9.1%)	13,753 (11.1%)
≥ 50	8958 (49.4%)	5285 (5%)	14,243 (11.5%)
Specific care supports (*n*, %)			
Vasopressor	7779 (42.9%)	1845 (1.7%)	9,624 (7.8%)
Median days [Q1–Q3]	3 [2–5]	2 [1–3]	2 [1–4]
Non-invasive ventilation	7785 (43%)	6944 (6.6%)	14,729 (11.9%)
Median days [Q1–Q3]	3 [2–6]	2 [1–4]	3 [1–5]
Invasive ventilation	7621 (42.1%)	712 (0.7%)	8,333 (6.7%)
Median days [Q1–Q3]	3 [2–8]	1 [1–4]	3 [2–7]
Length of stay in days (median [Q1–Q3])			
In the ICU	3 [1–7]	–	3 [1–7]*
In the IMCU	–	3 [2–5]	3 [2–5]**
In the critical care	5 [2–9]	3 [2–5]	3 [2–6]
In hospital	12 [5–22]	8 [4–14]	9 [4–15]
Mortality (*n*, %)			
In the critical care	6,423 (35.4%)	9,080 (8.6%)	15,503 (12.5%)
In hospital	7,541 (41.6%)	14,610 (13.8%)	22,151 (17.9%)

Patients admitted to both the ICU and IMCU during the same hospital stay were classified in the ICU group. IMCU refers to patients hospitalized in the IMCU without ICU stay(s) before and/or after the IMCU stay

Baseline characteristics of patients aged 80 years and older admitted to critical care during the study period are presented in Table 1. In IMCU, the most frequently used high-level organ support was non-invasive ventilation, administered to 6.6% of patients aged ≥ 80 years; other advanced organ supports were rarely used. In contrast, in ICU, high-level organ supports were frequently performed on patients aged 80 years and older, without major restriction compared with younger age classes (Table 1; Supplementary Table S5–S7). For example, invasive mechanical ventilation was used in 42.1% of ICU patients aged ≥ 80 years, compared with 46.8% and 49.1% in the 18–64 and 65–79-year age classes, respectively. Vasopressors were used in 42.9% of ICU patients aged ≥ 80 years, compared with 34.5% and 46.8% in the 18–64 and 65–79-year age classes, respectively (Table 1; Supplementary Table S5–S7).

In the population admitted to an ICU at least once, we then compared the utilization of IMCU according to the step-up trajectory and the step-down trajectory (as defined in Fig. 1). No major differences in the distribution of these patterns were observed across age classes (Fig. 2B). Overall, the step-up strategy was used in 12.4% of critically ill adults and 12.3% of patients aged 80 years and older. The step-down strategy was used in 25.0% of patients and in 23.1% of patients aged 80 years and older (Table 2, Supplementary Table S4).

**Table 2:** Characteristics of patients aged 80 years or older and admitted to the ICU in France, 2018, according to their trajectory in critical care units

	All *N* = 18,119 (100%)	With Step up *N* = 2,237 (12.3%)	Without Step up *N* = 15,882 (87.7%)	With Step down *N* = 4187 (23.1%)	Without Step down *N* = 13,932 (76.9%)
Men (*n*, %)	9571 (52.8%)	1300 (58.1%)	8271 (52.1%)	2200 (52.5%)	7371 (52.9%)
Age class (*n*, %)					
80–84 years old	9780 (54.0%)	1358 (60.7%)	8422 (53.0%)	2359 (56.3%)	7421 (53.3%)
85–89 years old	6290 (34.7%)	696 (31.1%)	5594 (35.2%)	1413 (33.7%)	4877 (35%)
≥ 90 years old	2049 (11.3%)	183 (8.2%)	1866 (11.7%)	415 (9.9%)	1634 (11.7%)
Mean ± std	84.7 ± 3.7	84.2 ± 3.4	84.8 ± 3.8	84.6 ± 3.6	84.6 ± 3.6
Median [Q1–Q3]	84 [82–87]	84 [81–86]	84 [81–87]	84 [82–87]	84 [82–87]
Charlson Comorbidity Index (*n*, %)					
Low 0	8651 (47.7%)	1162 (51.9%)	7489 (47.2%)	1984 (47.4%)	6667 (47.9%)
Middle [1, 2]	4504 (24.9%)	532 (23.8%)	3972 (25.0%)	1079 (25.8%)	3425 (24.6%)
High ≥ 3	4964 (27.4%)	543 (24.3%)	4421 (27.8%)	1124 (26.8%)	3840 (27.6%)
Median [Q1–Q3]	2 [1–3]	2 [1–3]	2 [1–3]	2 [1–3]	2 [1–3]
Hospital Frailty Risk Score (n, %)					
Low < 5	11,267 (62.2%)	1569 (70.1%)	9698 (61.1%)	2666 (63.7%)	8601 (61.7%)
Middle [5–15[	4,677 (25.8%)	483 (21.6%)	4,194 (26.4%)	1055 (25.2%)	3622 (26.0%)
High ≥ 15	2175 (12.0%)	185 (8.3%)	1990 (12.5%)	466 (11.1%)	1709 (12.3%)
Median [Q1–Q3]	6.1 [2.4–12.1]	4.7 [1.8–10]	6.3 [2.5–12.4]	5,7 [2.3–11.7]	5.7 [2.3–11.7]
Primary diagnosis registered in critical care (n, %)					
Respiratory	6738 (37.2%)	934 (41.8%)	5804 (36.5%)	1698 (40.6%)	5040 (36.2%)
Cardiovascular	4641 (25.6%)	596 (26.6%)	4045 (25.5%)	990 (23.6%)	3651 (26.2%)
Neurologic	2146 (11.8%)	226 (10.1%)	1920 (12.1%)	468 (11.2%)	1678 (12.0%)
Other	4594 (25.4%)	481 (21.5%)	4113 (25.9%)	1031 (24.6%)	3563 (25.6%)
SAPS II class (*n*, %)					
< 40	5035 (27.8%)	450 (20.1%)	4585 (28.9%)	1605 (38.3%)	3430 (24.6%)
[40–50[	4126 (22.8%)	519 (23.2%)	3607 (22.7%)	1117 (26.7%)	3009 (21.6%)
≥ 50	8958 (49.4%)	1268 (56.7%)	7690 (48.4%)	1465 (35.0%)	7493 (53.8%)
Specific care supports (*n*, %)					
Vasopressor	7779 (42.9%)	1242 (55.5%)	6537 (41.2%)	1463 (34.9%)	6316 (45.3%)
Median days [Q1–Q3]	3 [2–5]	6 [3–11]	2 [2–4]	6 [4–10]	2 [1–5]
Non-invasive ventilation	7785 (43.0%)	1125 (50.3%)	6660 (41.9%)	2194 (52.4%)	5591 (40.1%)
Median days [Q1–Q3]	3 [2–6]	4 [2–7]	3 [2–6]	4 [2–8]	3 [2–6]
Invasive ventilation	7621 (42.1%)	1202 (53.7%)	6419 (40.4%)	1290 (30.8%)	6331 (45.4%)
Median days [Q1–Q3]	3 [2–8]	5 [2–10]	3 [2–7]	5 [2–9]	3 [2–7]
Length of stay in days (median [Q1–Q3])					
In the ICU	3 [1–7]	5 [2–9]	3 [1–7]	4 [2–8]	3 [1–7]
In the IMCU	0 [0–1]	2 [1–6]	0 [0–0]	4 [2–8]	0 [0–0]
In the critical care	5 [2–9]	9 [4–16]	4 [2–9]	9 [5–16]	4 [1–7]
In hospital	12 [5–22]	17 [8–27]	12 [5–21]	18 [10–28]	11 [4–19]
Mortality (*n*, %)					
In the critical care	6423 (35.4%)	1043 (46.6%)	5380 (33.9%)	417 (10.0%)	6006 (43.1%)
In hospital	7541 (41.6%)	1176 (52.6%)	6365 (40.1%)	670 (16.0%)	6871 (49.3%)

“Step-up” refers to an IMCU stay before hospitalization in the ICU (IMCU → ICU and IMCU → ICU → IMCU). “Step-down” refers to an IMCU stay after hospitalization in the ICU (ICU → IMCU and IMCU → ICU → IMCU). The mixed IMCU → ICU → IMCU trajectory involved 632 patients aged 80 years or older

**Fig. 3:**
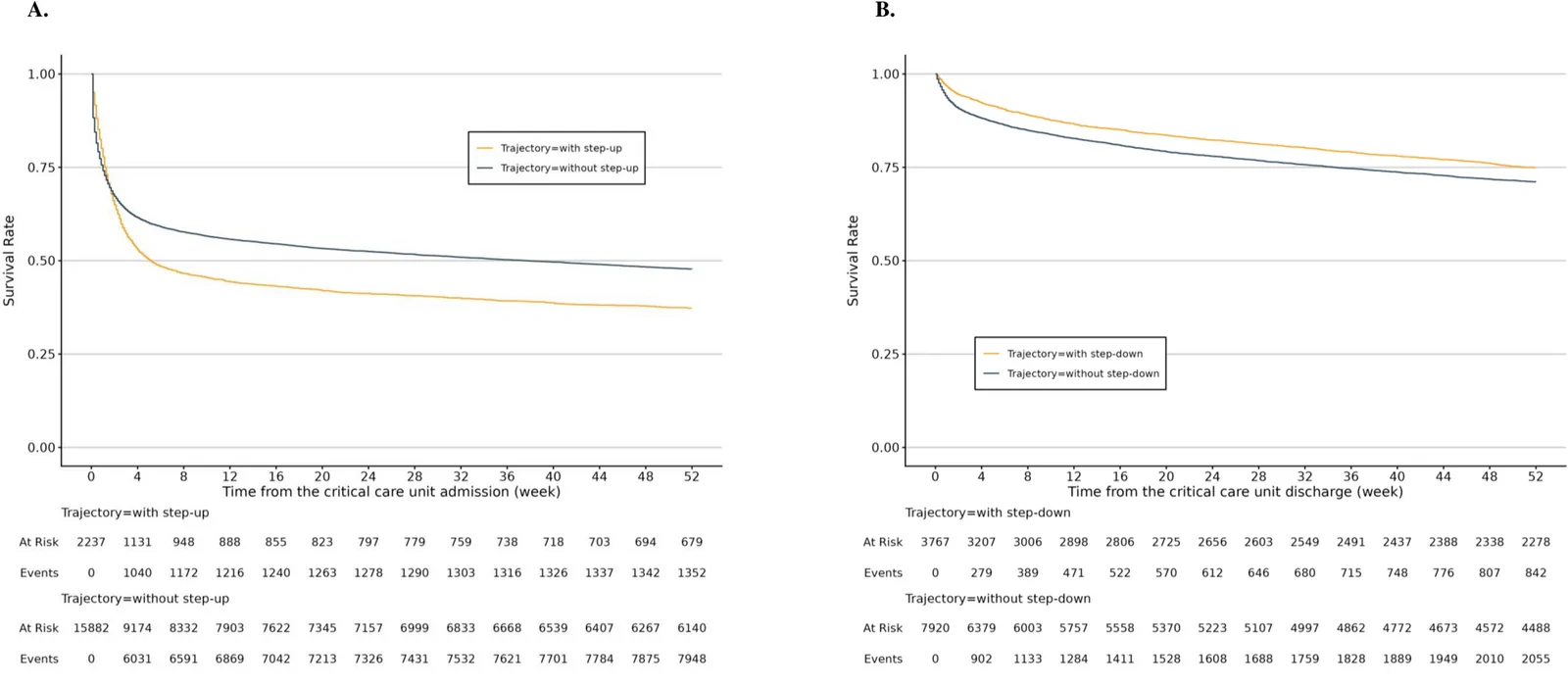
Kaplan–Meier curves showing the cumulative probabilities of survival, up to 12 months in France. **A** after ICU or IMCU admission according to the step-up strategy, **B** after critical care discharge according to the step-down strategy. *ICU* intensive care unit, *IMCU* intermediate care unit

The step-up strategy was associated with high mortality. Among patients aged ≥ 80 years, mortality in critical care was 33.9% for those admitted directly to ICU but increased to 46.6% in a step-up trajectory (Table 2). Kaplan–Meier curves presented in Fig. 3A indicated higher cumulative one-year mortality in the step-up group after critical care admission (ICU or IMCU). However, this simple interpretation is complicated by the early curve crossing, suggesting that the effect of step-up was time-dependent and not uniformly harmful. Patients managed with a step-up strategy were not older, did not have more comorbidities, and were not frailer than those admitted directly to ICU. However, they exhibited greater acute severity, as reflected by higher SAPS II scores and more frequent use of advanced organ supports (Table 2). The duration of IMCU stay before ICU transfer was short (median 2 [1–6] days).

Regarding the step-down strategy in patients aged ≥ 80 years, individuals managed with this trajectory had similar age, Charlson comorbidity index, and hospital frailty risk scores compared with patients exclusively taken care of in ICU (Table 2). However, acute severity was lower in step-down patients, as indicated by lower SAPS II scores. Notably, their management was less invasive, with more frequent use of non-invasive ventilation (52.4% vs. 39.3% in ICU patients without step-down) and reduced use of invasive ventilation and vasopressors (Table 2). The step-down strategy extended the overall length of stay in critical care for very old people (9 [4–16] days compared to 3 [1–7] days without step-down). Interpretation of mortality in the step-down group is complex, as it includes a selected population of patients discharged alive from ICU (Table 2). To avoid immortal time bias, we then restricted the analysis to patients alive at critical care discharge. Lower cumulative mortality was observed for patients discharged from critical care via a step-down strategy (Fig. 3B, Supplementary Table S8). One-year mortality for patients alive at critical care discharge was 31.48% when discharged directly from ICU and 27.04% when discharged with a step-down strategy, corresponding to an absolute risk reduction of 4.44 percentage points and a relative risk reduction of 14.10% (RR = 0.86).

The mixed IMCU-ICU-IMCU trajectory in patients aged ≥ 80 years involved a small number of patients (*n* = 632) complex to interpret and not analyzed further.

## Discussion

We characterized critical-care trajectories involving IMCU use among very old critically ill patients at a national French level over one year. We observed that the oldest age class was more frequently admitted to the IMCU, whereas ICU admission was more often preferred for younger adults. Interestingly, transition patterns involving the IMCU within the critical care pathway showed opposite associations with mortality among very old patients. A step-up strategy was associated with higher mortality, whereas a step-down strategy was associated with more favorable outcomes.

IMCU deployment varies widely across healthcare organizations, and IMCU availability has been reported to be associated with improved outcomes [30, 31]. Beyond availability, IMCU management strategies may also vary considerably. The step-down strategy, in which IMCU care follows an ICU stay, provides an intermediate level of care between the ICU and the general medical ward. In older critically ill patients, systematic promotion of ICU admission has not been shown to improve survival [32]; however, patients admitted to either an IMCU or an ICU demonstrated better survival compared with those admitted directly to a general hospital ward [33]. Patient trajectories—specifically step-up and step-down strategies between IMCU and ICU—have been less investigated. In a prospective multicenter observational cohort, a step-down strategy was associated with a trend toward lower mortality, and IMCU admission, regardless of the trajectory of admission (from the emergency department, post-ICU, or post-surgery), was associated with a statistically significant reduction in mortality [5]. Conversely, a step-up trajectory was associated with higher mortality compared with patients directly transferred from the emergency department to the ICU [34]. We observed a similar pattern in our cohort for the step-up trajectory, particularly for the older patients (IMCU to ICU). This finding may reflect several distinct clinical scenarios: deterioration despite appropriate initial treatment allocation, suggesting intrinsic disease severity; underestimation of initial disease severity; or misclassification of the appropriate level of care intensity, whereby patients initially deemed ineligible for ICU-level care were subsequently escalated to the ICU. Mortality among very old patients with prior IMCU admission was substantially higher than among those directly admitted to the ICU. The early crossing of the Kaplan–Meier curves indicated non-proportional hazards and may reflect time-dependent treatment effects. One possible explanation is that the initial severity assessment was misleading in this very old population. Older adults often live with comorbid conditions such as multimorbidity and frailty, which are associated with higher late mortality [35]. It is therefore plausible that the initiation of aggressive treatment was delayed, and that this delay contributed to the observed outcomes. Overall, these findings suggest that the step-up strategy more likely reflected an initial underestimation of acute severity at presentation rather than a deliberate triage decision.

The challenge of caring for older patients in critical care does not end at the ICU doors [13, 35]. Serious complications, including geriatric syndromes, in critically ill patients may appear during the transition out of intensive care. For instance, swallowing disorders and delirium are common among critically ill older patients and are associated with a cascade of adverse outcomes [35]. Oropharyngeal dysphagia has been reported in more than 80% of very old patients newly hospitalized and is significantly associated with respiratory infections [36]. Delirium has consistently been linked to increased mortality and prolonged hospital stays in older adults [37]. It is possible that a higher incidence of ICU-related geriatric syndromes, such as delirium or swallowing disorders, might support earlier ICU discharge because of a benefit–risk balance no longer in favor of prolonging the ICU stay, as underlying frailty is unmasked during the acute illness. This highlights the need to better define the optimal duration of intensive-level care in older adults. This observation has motivated the development of step-down strategies. IMCUs typically have a higher staff-to-patient ratio and are often better organized to manage the multiple complications that may arise immediately after ICU discharge in older adults. Moreover, IMCUs allow continuous vital monitoring that is not available in conventional wards. We therefore hypothesize that the favorable long-term outcomes observed with the step-down strategy may result from the continuous, comprehensive management enabled by IMCU organization during the transition out of intensive care and then contribute to improved outcomes. Finally, our study was designed to assess vital status and could not evaluate other potential benefits associated with the higher staff-to-patient ratio in IMCUs. For example, evidence from a recent systematic review suggested that low staff-to-patient ratios are associated with a greater risk of complications (e.g., nosocomial infections, reintubation, adverse events) and worse overall outcomes [38]. Similarly, early mobilization might be easier in wards with higher staff-to-patient ratios, and is clearly associated with improved short- and long-term functional outcomes after critical illness [39].

Defining the most appropriate level of care between ICU and IMCU remains a complex process, particularly in older adults. Despite a higher rate of IMCU admission among older patients, the comparable frequencies of step-up to ICU suggest that IMCU was not preferentially used as a transitional or preparatory stage before ICU admission in this population. These findings highlight the need for clearer guidelines and decision-support tools for ICU admission of older adults. While epidemiologic approaches are essential to describe global trajectories and patterns of care at the population level, they cannot fully capture the complexity of individual clinical situations. Decisions regarding ICU or IMCU admission should therefore not rely solely on aggregated data but must incorporate patient-centered factors such as frailty, comorbidities, functional status, goals of care, and expected benefit from intensive support. Indeed, advancing aging-focused critical care research requires complementary methodological approaches. Collaborative efforts involving geriatricians, epidemiologists, intensivists, emergency specialists, nurses, rehabilitation therapists, and social workers are therefore a top priority.

Some limits of the study should be underscored and discussed. First, using ICD-10-based administrative data, it was not possible to determine whether patients hospitalized in the IMCU had do-not-resuscitate orders and/or were denied ICU admission, as such information is not captured in hospital discharge summaries. Second, although French hospital administrative databases are powerful tools for identifying nationwide patterns, they are limited in their ability to support detailed, granular, patient-level analyses. Third, surgical patients were not included in this study because the prognosis for planned perioperative situations is already well-documented and differs significantly from acute organ dysfunction in very old patients [22]. Fourth, we selected the 2018 period because the COVID-19 pandemic disrupted usual conditions and post-pandemic care pathways had not yet returned to their pre-pandemic norms [4]. However, we previously demonstrated that the 2018 period was a representative period for trends in IMCU and ICU admissions in France [4]. Finally, because this study included the French population, the findings may not be readily generalizable to countries with different healthcare systems or differing IMCU and ICU admission policies.

In conclusion, using national health data we characterized critical-care trajectories in very old patients, and found that IMCUs play a central role in their management. One of the most notable findings was that a step-down strategy was associated with reduced mortality in critically ill very old patients. As the observational design of our study does not allow causal inference, prospective clinical trials are encouraged to evaluate the potential benefit of IMCU care for older adults transitioning out of intensive care.

## Supplementary Information

Below is the link to the electronic supplementary material.

Supplementary file1 (DOCX 64 KB)

## Data Availability

Restrictions apply to the availability of these data and so are not publicly available. However, aggregated data are available from the authors upon reasonable request and with the permission of the institution, or via the national Hub ATIH (https://www.atih.sante.fr/).
