# Supplementary material for: Patterns of medical intermediate care unit use in critically ill very old patients in France
Source: Eur Geriatr Med. 2026 May 13;17(4):1793–802. doi: 10.1007/s41999-026-01495-x (PMC13562155; doi:10.1007/s41999-026-01495-x)
Supplement: Supplementary file 1 [file 41999_2026_1495_MOESM1_ESM.docx]

**Supplementary materials: Tables**

**Table S1.** Organ classification of ICD-10 primary diagnosis.

**Table S2.** French CPT codes of specifics ICU care supports.

**Table S3.** French healthcare unit specific authorization of ICU and IMCU

**Table S4.** Characteristics of all patients aged 18 years or older hospitalized in critical care.

**Table S5.** Characteristics of patients aged 18 to 64 years hospitalized in critical care units.

**Table S6.** Characteristics of patients aged 65 to 79 years hospitalized in critical care units.

**Table S7.** Characteristics of patients aged 80 years or older hospitalized in critical care units.

**Table S8.** Characteristics of patients aged 80 years or older and admit in ICU and alive at critical care discharge, according to the “Step-down” trajectory

**Table S1.** Organ classification of ICD-10 primary diagnosis.

| **ICD-10 classification by organ** | **List of ICD-10 codes** |
| --- | --- |
| CARDiOVASCULAR | A021, A327-A329, A392-A395, A40-A41, A427, A440, A482-A484, A75-A79, A90-A99, B332, B334, B377, B464, B49, B570, B572, E105, E115, E86, I00-I25, I30-I52, I70-I71, I720-I721, I723-I729, I74, I77, I79-I80, I821-I822, I828-I829, I83, I95, I970-I971, I978-I979, I980-I981, I983-I988, I99, J853, J853, L97, O08-O14, O16, O22, O72, O751, O85, O871, O88, O903, O990, Q20-Q28, R00-R01, R03, R55, R57-R58, R60, R65, R680, R931, R943, T673, T68, T78, T811, R60, R65, R680, R931, R943, T673, T68 |
| RESPIRATORY | A15-A16, A19, A310, A36-A38, A420, A430, A481, A691, A70, B012, B052, B250, B342, B371, B44, B450, B460, B510, B55, B573, B583, B59, E662, E840, I26-28,  J00-J84, J850-J852, J86-J99, K12-K14, Q30-Q37,  R04-R09, R942, T59, T71, T751, U04, Z930, Z942, Z991 |
| NEUROLOGY | A17, A321, A390, A521-A523, A80-A89, B003-B004, B010-B011, B020-B023, B050-B051, B261-B262, B375-B376, B451, B457, B461, B500, B56, B571, B574, B582, B690, E100, E104, E110, E113, E116, E120, E130, E134, E140, E144, E15, G00-G99, H34, H48-H54, H81-H91,I60-I69, O150-O159, O745, O873, Q03, R20, R25-R29, R40-R49, R51-R52, R54, R56, R90, R941, T58, T671, Z033, Z036, Z982 |

*International Classification of Diseases (ICD-10)*

**Table S2:** French classification of procedures terminology (CPT) for specific ICU care supports.

| **CPT procedure** | **Codes** | **Labels** |
| --- | --- | --- |
| Vasopressor | EQLF001 | Continuous intravenous injection of dobutamine or dopamine at a rate of less than 8 micrograms per kilogram per minute [µg/kg/min], or dopexamine outside the neonatal period, per 24 hours |
|  | EQLF003 | Continuous intravenous injection of dobutamine or dopamine at a rate exceeding 8 micrograms per kilogram per minute [µg/kg/min], adrenaline or noradrenaline outside the neonatal period, per 24 hours |
| Non-Invasive mechanical ventilation CPAP | GLLD003 | Spontaneous ventilation by face mask, nasal cannula or nasopharyngeal tube, without inspiratory support, with positive expiratory pressure [VS-PEP] [Continuous positive airway pressure] [CPAP], per 24 hours |
| Non-Invasive mechanical ventilation BPAP | GLLD019 | Non-invasive barometric or volumetric ventilation by face mask for at least 2 hours cumulative over a 12-hour period, for acute respiratory failure |
|  | GLLD012 | Continuous mechanical ventilation with face mask for ventilatory support, per 24 hours |
| Invasive mechanical ventilation | GLLD015 | Intratracheal mechanical ventilation with positive expiratory pressure [PEEP] less than or equal to 6 and FiO2 less than or equal to 60%, per 24 hours |
|  | GLLD008 | Intratracheal mechanical ventilation with positive expiratory pressure [PEEP] greater than 6 and/or FiO2 greater than 60%, per 24 hours |
|  | GLLD004 | Intratracheal mechanical ventilation with positive expiratory pressure [PEEP] greater than 6 and/or FiO2 greater than 60%, with alternate prone positions every 24 hours |

**Table S3.** French healthcare unit specific authorization of ICU and IMCU

| **Type of unit** | **Code of medical unit** | **Labels** |
| --- | --- | --- |
| ICU | 01A | Adult intensive care, excluding severe burns ICU |
| IMCU | 02A | Intensive cardiac care = CCU (Cardiac Care Unit) |
|  | 02B | Other intensive care (excluding Stroke Units, CCU, and Neonatology) |
|  | 03A | Adult intermediate care, excluding severe burns intermediate care unit |
|  | 16 | Hematology unit with an air treatment system |
|  | 18 | Intensive care in Stroke Unit |

*Intensive Care Unit (ICU)*

*InterMediate Care Unit (IMCU)*

**Table S4.** Characteristics of all patients aged 18 years or older hospitalized in critical care.

|  | **ICU only** | **IMCU-ICU** | **IMCU-ICU-IMCU** | **ICU-IMCU** | **IMCU only** | **All** |
| --- | --- | --- | --- | --- | --- | --- |
|  | N=67,359 (15.3%) | N=8,220 (1.9%) | N=4,238 (1.0%) | N=20,996 (4.8%) | N=338,400 (77.0%) | N=439,213 (100%) |
| **Men (n, %)** | 40,261 (59.8%) | 5,226 (63.6%) | 2,709 (63.9%) | 12,661 (60.3%) | 187,337 (55.4%) | 248,194 (56.5%) |
| **Age class (n, %)** |  |  |  |  |  |  |
| 18-64 y.o. | 32,040 (47.6%) | 3,239 (39.4%) | 1,923 (45.4%) | 10,028 (47.8%) | 122,972 (36.3%) | 170,202 (38.8%) |
| 65-79 y.o. | 22,992 (34.1%) | 3,376 (41.1%) | 1,683 (39.7%) | 7,413 (35.3%) | 109,855 (32.5%) | 145,319 (33.1%) |
| ≥ 80 y.o. | 12,327 (18.3%) | 1,605 (19.5%) | 632 (14.9%) | 3,555 (16.9%) | 105,573 (31.2%) | 123,692 (28.2%) |
| Mean ± std | 62.8 ± 17.6 | 66.0 ± 15.2 | 63.8 ± 15.6 | 62.7 ± 17.1 | 67.9 ± 17.8 | 66.8 ± 17.8 |
| Median [Q1-Q3] | 66 [52-76] | 68 [58-77] | 66 [55-75] | 65 [52-76] | 71 [57-82] | 70 [56-81] |
| **Charlson Comorbidity Index (n, %)** | |  |  |  |  |  |
| Low 0 | 36,887 (54.8%) | 4,337 (52.8%) | 2,331 (55%) | 11,676 (55.6%) | 209,880 (62%) | 265,111 (60.4%) |
| Middle [1-2] | 14,470 (21.5%) | 1,830 (22.3%) | 930 (21.9%) | 4,738 (22.6%) | 65,377 (19.3%) | 87,345 (19.9%) |
| High ≥ 3 | 16,002 (23.8%) | 2,053 (25%) | 977 (23.1%) | 4,582 (21.8%) | 63,143 (18.7%) | 86,757 (19.8%) |
| Median [Q1-Q3] | 2 [0-4] | 2 [1-4] | 2 [1-4] | 2 [0-3] | 2 [0-3] | 2 [0-3] |
| **Primary diagnosis registered in critical care (n, %)** | | |  |  |  |  |
| Respiratory | 22,112 (32.8%) | 3,183 (38.7%) | 1,824 (43.0%) | 7,942 (37.8%) | 58,948 (17.4%) | 94,009 (21.4%) |
| Cardiovascular | 12,626 (18.7%) | 1,849 (22.5%) | 812 (19.2%) | 3,513 (16.7%) | 109,183 (32.3%) | 127,983 (29.1%) |
| Neurologic | 8,460 (12.6%) | 975 (11.9%) | 472 (11.1%) | 2,549 (12.1%) | 78,425 (23.2%) | 90,881 (20.7%) |
| Other | 24,161 (35.9%) | 2,213 (26.9%) | 1,130 (26.7%) | 6,992 (33.3%) | 91,844 (27.1%) | 126,340 (28.8%) |
| **SAPSII class (n, %)** |  |  |  |  |  |  |
| < 40 | 30,493 (45.3%) | 2,612 (31.8%) | 1,972 (46.5%) | 11,532 (54.9%) | 312,336 (92.3%) | 358,945 (81.7%) |
| [40-50[ | 11,305 (16.8%) | 1,531 (18.6%) | 992 (23.4%) | 3,989 (19%) | 17,051 (5%) | 34,868 (7.9%) |
| ≥ 50 | 25,561 (37.9%) | 4,077 (49.6%) | 1,274 (30.1%) | 5,475 (26.1%) | 9,013 (2.7%) | 45,400 (10.3%) |
| **Specific care supports (n, %)** | |  |  |  |  |  |
| Vasopressor | 26,703 (39.6%) | 4,865 (59.2%) | 1,909 (45.0%) | 7,187 (34.2%) | 4,449 (1.3%) | 45,113 (10.3%) |
| Median days [Q1-Q3] | 2 [1-4] | 3 [2-6] | 3 [2-6] | 3 [2-5] | 2 [1-3] | 3 [1-5] |
| Non-invasive ventilation | 20,868 (31.0%) | 3,517 (42.8%) | 2,166 (51.1%) | 8,718 (41.5%) | 16,913 (5.0%) | 52,182 (11.9%) |
| Median days [Q1-Q3] | 3 [2-6] | 3 [2-6] | 4 [2-8] | 4 [2-7] | 2 [1-5] | 3 [1-6] |
| Invasive ventilation | 31,655 (47.0%) | 5,184 (63.1%) | 2,012 (47.5%) | 8,269 (39.4%) | 2,402 (0.7%) | 49,522 (11.3%) |
| Median days [Q1-Q3] | 3 [2-8] | 5 [2-12] | 7 [3-14.5] | 5 [2-12] | 2 [1-4] | 4 [2-9] |
| **Length of stay in days** (median [Q1-Q3]) | |  |  |  |  |  |
| In the ICU | 3 [1-7] | 5 [2-11] | 5 [2-11] | 4 [1-8] | / | 3 [1-8]* |
| In the IMCU | / | 1 [0-3] | 7 [4-13] | 3 [2-7] | 3 [1-5] | 3 [1-5]** |
| In the critical care | 3 [1-7] | 7 [3-15] | 14 [8-26] | 8 [4-15] | 3 [1-5] | 3 [1-5] |
| In hospital | 9 [3-17] | 15 [7-27] | 25 [14-41] | 16 [9-27] | 6 [3-12] | 7 [3-14] |
| **Death during the stay (n, %)** | |  |  |  |  |  |
| In the critical care | 18,523 (27.5%) | 3,769 (45.9%) | 325 (7.7%) | 905 (4.3%) | 15,201 (4.5%) | 38,723 (8.8%) |
| In the hospital stay | 20,640 (30.6%) | 4,056 (49.3%) | 478 (11.3%) | 1,496 (7.1%) | 24,150 (7.1%) | 50,820 (1.,6%) |

** Excluding patients without ICU*

*** Excluding patients without IMCU*

**Table S5.** Characteristics of patients aged 18 to 64 years hospitalized in critical care units.

|  | **ICU only** | **IMCU-ICU** | **IMCU-ICU-IMCU** | **ICU-IMCU** | **IMCU only** | **All** |
| --- | --- | --- | --- | --- | --- | --- |
|  | N=32,040 (18.8%) | N=3,239 (1.9%) | N=1,923 (1.1%) | N=10,028 (5.9%) | N=122,972 (72.3%) | N=170,202 (100%) |
| **Men (n, %)** | 19,521 (60.9%) | 2,076 (64.1%) | 1,228 (63.9%) | 6,206 (61.9%) | 74,553 (60.6%) | 103,584 (60.9%) |
| **Age** (y.o.) |  |  |  |  |  |  |
| Mean ± std | 48 ± 12.9 | 51 ± 11.9 | 50.2 ± 11.9 | 48.4 ± 12.7 | 48.5 ± 12.6 | 48.5 ± 12.7 |
| Median [Q1-Q3] | 51 [39-59] | 55 [45-60] | 54 [44-60] | 52 [40-59] | 52 [40-59] | 52 [40-59] |
| **Charlson Comorbidity Index (n, %)** | | |  |  |  |  |
| Low 0 | 20,807 (64.9%) | 1,945 (60%) | 1,172 (60.9%) | 6,578 (65.6%) | 90,297 (73.4%) | 120,799 (71%) |
| Middle [1-2] | 5,672 (17.7%) | 623 (19.2%) | 403 (21%) | 1,808 (18%) | 17,906 (14.6%) | 26,412 (15.5%) |
| High ≥ 3 | 5,561 (17.4%) | 671 (20.7%) | 348 (18.1%) | 1,642 (16.4%) | 14,769 (12%) | 22,991 (13.5%) |
| Median [Q1-Q3] | 1 [0-3] | 2 [0-4] | 2 [0-3] | 1 [0-3] | 1 [0-3] | 1 [0-3] |
| **Primary diagnosis registered in critical care (n, %)** | | |  |  |  |  |
| Respiratory | 9,037 (28.2%) | 1,178 (36.4%) | 786 (40.9%) | 3,436 (34.3%) | 21,959 (17.9%) | 36,396 (21.4%) |
| Cardiovascular | 4,398 (13.7%) | 549 (16.9%) | 313 (16.3%) | 1,235 (12.3%) | 30,050 (24.4%) | 36,545 (21.5%) |
| Neurologic | 4,214 (13.2%) | 424 (13.1%) | 248 (12.9%) | 1,325 (13.2%) | 28,374 (23.1%) | 34,585 (20.3%) |
| Other | 14,391 (44.9%) | 1,088 (33.6%) | 576 (30.0%) | 4,032 (40.2%) | 42,589 (34.6%) | 62,676 (36.8%) |
| **SAPSII class (n, %)** |  |  |  |  |  |  |
| < 40 | 19,020 (59.4%) | 1,495 (46.2%) | 1,181 (61.4%) | 6,663 (66.4%) | 119,885 (97.5%) | 148,244 (87.1%) |
| [40-50[ | 4,353 (13.6%) | 537 (16.6%) | 328 (17.1%) | 1,426 (14.2%) | 2,035 (1.7%) | 8,679 (5.1%) |
| ≥ 50 | 8,667 (27.1%) | 1,207 (37.3%) | 414 (21.5%) | 1,939 (19.3%) | 1,052 (0.9%) | 13,279 (7.8%) |
| **Specific care supports (n, %)** |  |  |  |  |  |  |
| Vasopressor | 10,636 (33.2%) | 1,716 (53.0%) | 792 (41.2%) | 3,135 (31.3%) | 1,102 (0.9%) | 17,381 (10.2%) |
| Median days [Q1-Q3] | 2 [1-4] | 3 [2-6] | 3 [2-6] | 3 [2-5] | 2 [1-3] | 2 [1-5] |
| Non-invasive ventilation | 7,572 (23.6%) | 1,238 (38.2%) | 882 (45.9%) | 3,319 (33.1%) | 4,120 (3.4%) | 17,131 (10.1%) |
| Median days [Q1-Q3] | 3 [1-5] | 3 [2-6] | 3 [2-7] | 4 [2-7] | 3 [1-5] | 3 [1-6] |
| Invasive ventilation | 14,850 (46.3%) | 2,016 (62.2%) | 959 (49.9%) | 4,276 (42.6%) | 854 (0.7%) | 22,955 (13.5%) |
| Median days [Q1-Q3] | 3 [2-7] | 5 [2-12] | 7 [3-16] | 5 [2-12] | 2 [1-4] | 3 [2-9] |
| **Length of stay in days** |  |  |  |  |  |  |
| In the ICU (median [Q1-Q3]) | 3 [1-6] | 5 [2-11] | 5 [2-12] | 3 [1-8] | / | 3 [1-7]* |
| In the IMCU (median [Q1-Q3]) | / | 1 [0-3] | 7 [4-14] | 3 [2-6] | 2 [1-4] | 2 [1-4]** |
| In the critical care (median [Q1-Q3]) | 3 [1-6] | 7 [3-14] | 14 [8-28] | 7 [4-15] | 2 [1-4] | 3 [1-5] |
| In hospital (median [Q1-Q3]) | 7 [2-15] | 14 [7-26] | 24 [13-43] | 13 [7-25] | 5 [2-9] | 5 [2-11] |
| **Mortality (n, %)** |  |  |  |  |  |  |
| In the critical care | 5,825 (18.2%) | 1,103 (34.1%) | 64 (3.3%) | 201 (2.0%) | 1 ,906 (1.5%) | 9,099 (5.3%) |
| In hospital | 6,300 (19.7%) | 1,159 (35.8%) | 97 (5.0%) | 313 (3.1%) | 2,900 (2.4%) | 10,769 (6.3%) |

** excluding patients without ICU*

*** excluding patients without IMCU*

**Table S6.** Characteristics of patients aged 65 to 79 years hospitalized in critical care units.

|  | **ICU only** | **IMCU-ICU** | **IMCU-ICU-IMCU** | **ICU-IMCU** | **IMCU only** | **All** |
| --- | --- | --- | --- | --- | --- | --- |
|  | N=22,992 (15.8%) | N=3,376 (2.3%) | N=1,683 (1.2%) | N=7,413 (5.1%) | N=109,855 (75.6%) | N=145,319 (100%) |
| **Men (n, %)** | 14,303 (62.2%) | 2,216 (65.6%) | 1,115 (66.3%) | 4,621 (62.3%) | 65,923 (60%) | 88,178 (60.7%) |
| **Age** (y.o.) |  |  |  |  |  |  |
| Mean ± std | 71.7 ± 4.3 | 71.8 ± 4.3 | 71.6 ± 4.2 | 71.6 ± 4.3 | 72 ± 4.3 | 71.9 ± 4.3 |
| Median [Q1-Q3] | 71 [68-75] | 72 [68-75] | 71 [68-75] | 71 [68-75] | 72 [68-76] | 72 [68-76] |
| **Charlson Comorbidity Index (n, %)** | |  |  |  |  |  |
| Low 0 | 10,250 (44.6%) | 1,555 (46.1%) | 834 (49.6%) | 3,439 (46.4%) | 63,560 (57.9%) | 79,638 (54.8%) |
| Middle [1-2] | 5,756 (25%) | 824 (24.4%) | 378 (22.5%) | 2,000 (27%) | 22,870 (20.8%) | 31,828 (21.9%) |
| High ≥ 3 | 6,986 (30.4%) | 997 (29.5%) | 471 (28%) | 1,974 (26.6%) | 23,425 (21.3%) | 33,853 (23.3%) |
| Median [Q1-Q3] | 2 [1-4] | 2 [1-4] | 2 [1-4] | 2 [1-4] | 2 [0-4] | 2 [1-4] |
| **Primary diagnosis registered in critical care (n, %)** | | |  |  |  |  |
| Respiratory | 8,672 (37.7%) | 1,368 (40.5%) | 741 (44.0%) | 3,105 (41.9%) | 19,446 (17.7%) | 33,332 (22.9%) |
| Cardiovascular | 5,033 (21.9%) | 844 (25.0%) | 359 (21.3%) | 1,428 (19.3%) | 35,882 (32.7%) | 43,546 (30.0%) |
| Neurologic | 2,746 (11.9%) | 373 (11.0%) | 176 (10.5%) | 804 (10.8%) | 26,773 (24.4%) | 30,872 (21.2%) |
| Other | 6,541 (28.4%) | 791 (23.4%) | 407 (24.2%) | 2,076 (28.0%) | 27,754 (25.3%) | 37,569 (25.9%) |
| **SAPSII class (n, %)** |  |  |  |  |  |  |
| < 40 | 8,315 (36.2%) | 845 (25%) | 613 (36.4%) | 3,442 (46.4%) | 101,790 (92.7%) | 115,005 (79.1%) |
| [40-50[ | 4,259 (18.5%) | 678 (20.1%) | 461 (27.4%) | 1,649 (22.2%) | 5,389 (4.9%) | 12,436 (8.6%) |
| ≥ 50 | 10,418 (45.3%) | 1,853 (54.9%) | 609 (36.2%) | 2,322 (31.3%) | 2,676 (2.4%) | 17,878 (12.3%) |
| **Specific care supports (n, %)** | | |  |  |  |  |
| Vasopressor | 10,732 (46.7%) | 2,168 (64.2%) | 856 (50.9%) | 2,850 (38.4%) | 1,502 (1.4%) | 18,108 (12.5%) |
| Median days [Q1-Q3] | 3 [2-5] | 3 [2-6] | 3 [2-6] | 3 [2-5] | 2 [1-3] | 3 [2-5] |
| Non-invasive ventilation | 8,453 (36.8%) | 1,531 (45.3%) | 907 (53.9%) | 3,582 (48.3%) | 5,849 (5.3%) | 20,322 (14.0%) |
| Median days [Q1-Q3] | 3 [2-6] | 3 [2-7] | 4 [2-8] | 4 [2-8] | 3 [1-5] | 3 [2-6] |
| Invasive ventilation | 11,427 (49.7%) | 2,215 (65.6%) | 804 (47.8%) | 2,952 (39.8%) | 836 (0.8%) | 18,234 (12.5%) |
| Median days [Q1-Q3] | 4 [2-9] | 5 [2-12] | 7 [3-14] | 6 [3-12] | 2 [1-4] | 4 [2-10] |
| **Length of stay in days** |  |  |  |  |  |  |
| In the ICU (median [Q1-Q3]) | 4 [2-8] | 5 [2-12] | 5 [3-11] | 4 [2-9] | / | 4 [2-9]* |
| In the IMCU (median [Q1-Q3]) | / | 1 [0-3] | 7 [4-14] | 4 [2-7] | 3 [1-5] | 3 [1-5]** |
| In the critical care (median [Q1-Q3]) | 4 [2-8] | 8 [4-16] | 15 [9-25] | 9 [5-16] | 3 [1-5] | 3 [2-6] |
| In hospital (median [Q1-Q3]) | 11 [4-20] | 16 [7-29] | 26 [16-41] | 18 [10-30] | 7 [3-12] | 8 [4-15] |
| **Mortality (n, %)** |  |  |  |  |  |  |
| In the critical care | 7,640 (33.2%) | 1,718 (50.9%) | 166 (9.9%) | 382 (5.2%) | 4,215 (3.8%) | 14,121 (9.7%) |
| In hospital | 8,510 (37.0%) | 1,856 (55.0%) | 246 (14.6%) | 648 (8.7%) | 6,640 (6.0%) | 17,900 (12.3%) |

** excluding patients without ICU*

*** excluding patients without IMCU*

**Table S7.** Characteristics of patients aged 80 years or older hospitalized in critical care units.

|  | **ICU** | **IMCU-ICU** | **IMCU-ICU-IMCU** | **ICU-IMCU** | **IMCU** | **All** |
| --- | --- | --- | --- | --- | --- | --- |
|  | N=12,327 (10.0%) | N=1,605 (1.3%) | N=632 (0.5%) | N=3,555 (2.9%) | N=105,573 (85.4%) | N=123,692 (100%) |
| **Men (n, %)** | 6,437 (52.2%) | 934 (58.2%) | 366 (57.9%) | 1,834 (51.6%) | 46,861 (44.4%) | 56,432 (45.6%) |
| **Age class (n, %)** |  |  |  |  |  |  |
| 80-84 y.o. | 6,447 (52.3%) | 974 (60.7%) | 384 (60.8%) | 1,975 (55.6%) | 42,323 (40.1%) | 52,103 (42.1%) |
| 85-89 y.o. | 4,374 (35.5%) | 503 (31.3%) | 193 (30.5%) | 1,220 (34.3%) | 39,282 (37.2%) | 45,572 (36.8%) |
| ≥ 90 y.o. | 1,506 (12.2%) | 128 (8%) | 55 (8.7%) | 360 (10.1%) | 23,968 (22.7%) | 26,017 (21%) |
| Mean ± std | 84.9 ± 3.8 | 84.1 ± 3.4 | 84.2 ± 3.5 | 84.6 ± 3.6 | 86.2 ± 4.4 | 86 ± 4.3 |
| Median [Q1-Q3] | 84 [82-87] | 84 [82-86] | 84 [81-86] | 84 [82-87] | 86 [83-89] | 85 [83-89] |
| **Charlson Comorbidity Index (n, %)** | |  |  |  |  |  |
| Low 0 | 5,830 (47.3%) | 837 (52.1%) | 325 (51.4%) | 1,659 (46.7%) | 56,023 (53.1%) | 64,674 (52.3%) |
| Middle [1-2] | 3,042 (24.7%) | 383 (23.9%) | 149 (23.6%) | 930 (26.2%) | 24,601 (23.3%) | 29,105 (23.5%) |
| High ≥ 3 | 3,455 (28%) | 385 (24%) | 158 (25%) | 966 (27.2%) | 24,949 (23.6%) | 29,913 (24.2%) |
| Median [Q1-Q3] | 2 [1-3] | 2 [1-3] | 2 [1-3] | 2 [1-3] | 2 [0-3] | 2 [1-3] |
| **Hospital Frailty Risk Score (n, %)** | |  |  |  |  |  |
| Low < 5 | 7,468 (60.6%) | 1,133 (70.6%) | 436 (69%) | 2,230 (62.7%) | 69,204 (65.6%) | 80,471 (65.1%) |
| Middle [5-15[ | 3,280 (26.6%) | 342 (21.3%) | 141 (22.3%) | 914 (25.7%) | 24,464 (23.2%) | 29,141 (23.6%) |
| High ≥ 15 | 1,579 (12.8%) | 130 (8.1%) | 55 (8.7%) | 411 (11.6%) | 11,905 (11.3%) | 14,080 (11.4%) |
| Median [Q1-Q3] | 6.3 [2.5-12.5] | 4.6 [1.8-9.9] | 4.7 [1.8-10.4] | 5.9 [2.3-11.85] | 5.8 [2.1-12.1] | 5.9 [2.2-12.1] |
| **Primary diagnosis registered in critical care (n, %)** | | |  |  |  |  |
| Respiratory | 4,403 (35.7%) | 637 (39.7%) | 297 (47.0%) | 1,401 (39.4%) | 17,543 (16.6%) | 24,281 (19.6%) |
| Cardiovascular | 3,195 (25.9%) | 456 (28.4%) | 140 (22.2%) | 850 (23.9%) | 43,251 (41.0%) | 47,892 (38.7%) |
| Neurologic | 1,500 (12.2%) | 178 (11.1%) | 48 (7.6%) | 420 (11.8%) | 23,278 (22.0%) | 25,424 (20.6%) |
| Other | 3,229 (26.2%) | 334 (20.8%) | 147 (23.3%) | 884 (24.9%) | 21,501 (20.4%) | 26,095 (21.1%) |
| **SAPS II class (n, %)** |  |  |  |  |  |  |
| < 40 | 3,158 (25.6%) | 272 (16.9%) | 178 (28.2%) | 1,427 (40.1%) | 90,661 (85.9%) | 95,696 (77.4%) |
| [40-50[ | 2,693 (21.8%) | 316 (19.7%) | 203 (32.1%) | 914 (25.7%) | 9,627 (9.1%) | 13,753 (11.1%) |
| ≥ 50 | 6,476 (52.5%) | 1,017 (63.4%) | 251 (39.7%) | 1,214 (34.1%) | 5,285 (5%) | 14,243 (11.5%) |
| **Specific care supports (n, %)** | |  |  |  |  |  |
| Vasopressor | 5,335 (43.3%) | 981 (61.1%) | 261 (41.3%) | 1,202 (33.8%) | 1845 (1.7%) | 9,624 (7.8%) |
| Median days [Q1-Q3] | 2 [1-4] | 3 [2-6] | 3 [2-5] | 3 [2-5] | 2 [1-3] | 2 [1-4] |
| Non-invasive ventilation | 4,843 (39.3%) | 748 (46.6%) | 377 (59.7%) | 1,817 (51.1%) | 6,944 (6.6%) | 14,729 (11.9%) |
| Median days [Q1-Q3] | 3 [2-5] | 3 [2-6] | 4 [2-9] | 4 [2-8] | 2 [1-4] | 3 [1-5] |
| Invasive ventilation | 5,378 (43.6%) | 953 (59.4%) | 249 (39.4%) | 1,041 (29.3%) | 712 (0.7%) | 8,333 (6.7%) |
| Median days [Q1-Q3] | 3 [2-7] | 4 [2-10] | 5 [3-11] | 5 [2-9] | 1 [1-4] | 3 [2-7] |
| **Length of stay in days** (median [Q1-Q3]) | |  |  |  |  |  |
| In the ICU | 3 [1-7] | 5 [2-9] | 5 [2-9] | 4 [1-7] | / | 3 [1-7]* |
| In the IMCU | / | 1 [0-3] | 7 [4-13] | 4 [2-7] | 3 [2-5] | 3 [2-5]** |
| In the critical care | 3 [1-7] | 7 [3-13] | 14 [8-22] | 8 [5-15] | 3 [2-5] | 3 [2-6] |
| In hospital | 10 [4-19] | 14 [6-24] | 24 [15-36] | 17 [11-27] | 8 [4-14] | 9 [4-15] |
| **Mortality (n, %)** |  |  |  |  |  |  |
| In the critical care | 5,058 (41.0%) | 948 (59.1%) | 95 (15.0%) | 322 (9.1%) | 9,080 (8.6%) | 15,503 (12.5%) |
| In hospital | 5,830 (47.3%) | 1,041 (64.9%) | 135 (21.4%) | 535 (15.0%) | 14,610 (13.8%) | 22,151 (17.9%) |

** excluding patients without ICU (IMCU only)*

*** excluding patients without IMCU (ICU only)*

**Table S8. Characteristics of patients aged 80 years or older and admit in ICU and alive at critical care discharge, according to the** “Step-down” trajectory (ICU→IMCU and IMCU→ICU→IMCU). The mixed IMCU→ICU→IMCU trajectory involved 537 patients.

|  | **All patients aged 80 years or older in ICU**  **alive at critical care discharge** | **With**  **Step down**  **alive at critical care discharge** | **Without**  **Step down**  **alive at critical care discharge** |
| --- | --- | --- | --- |
|  | N=11,696 | N= 3,770 (32.2%) | N=7,926 (67.8%) |
| **Men (n, %)** | 6,083 (52.0%) | 1,968 (52.2%) | 4,115 (51.9%) |
| **Age class (n, %)** |  |  |  |
| 80-84 y.o. | 6,381 (54.6%) | 2,148 (57%) | 4,233 (53.4%) |
| 85-89 y.o. | 4,040 (34.5%) | 1,272 (33.7%) | 2,768 (34.9%) |
| ≥ 90 y.o. | 1,275 (10.9%) | 350 (9.3%) | 925 (11.7%) |
| Mean ± std | 84.7 ± 3.7 | 84.5 ± 3.6 | 84.8 ± 3.8 |
| Median [Q1-Q3] | 84 [82-87] | 84 [82-87] | 84 [82-87] |
| **Charlson Comorbidity Index (n, %)** |  |  |  |
| Low 0 | 5,535 (47.3%) | 1,803 (47.8%) | 3,732 (47.1%) |
| Middle [1-2] | 2,937 (25.1%) | 970 (25.7%) | 1,967 (24.8%) |
| High ≥ 3 | 3,224 (27.6%) | 997 (26.4%) | 2,227 (28.1%) |
| Median [Q1-Q3] | 2 [1-3] | 2 [1-3] | 2 [1-3] |
| **Hospital Frailty Risk Score (n, %)** |  |  |  |
| Low < 5 | 7,292 (62.3%) | 2,415 (64.1%) | 4,877 (61.5%) |
| Middle [5-15[ | 2,980 (25.5%) | 939 (24.9%) | 2,041 (25.8%) |
| High ≥ 15 | 1,424 (12.2%) | 416 (11%) | 1,008 (12.7%) |
| Median [Q1-Q3] | 6.1 [2.3-12.2] | 5.7 [2.3-11.5] | 6.3 [2.4-12.5] |
| **Primary diagnosis registered**  **in critical care (n, %)** |  |  |  |
| Respiratory | 4,568 (39.1%) | 1,532 (40.6%) | 3,036 (38.3%) |
| Cardiovascular | 2,627 (22.5%) | 872 (23.1%) | 1,755 (22.1%) |
| Neurologic | 1,100 (9.4%) | 420 (11.1%) | 680 (8.6%) |
| Other | 3,401 (29.1%) | 946 (25.1%) | 2,455 (31.0%) |
| **SAPS II class (n. %)** |  |  |  |
| < 40 | 4.381 (37.5%) | 1.485 (39.4%) | 2.896 (36.5%) |
| [40-50[ | 3.306 (28.3%) | 1.012 (26.8%) | 2.294 (28.9%) |
| ≥ 50 | 4.009 (34.3%) | 1.273 (33.8%) | 2.736 (34.5%) |
| **Specific care supports (n. %)** |  |  |  |
| Vasopressor | 3.652 (31.2%) | 1.294 (34.3%) | 2.358 (29.8%) |
| Median days [Q1-Q3] | 3 [2-4] | 3 [2-5] | 3 [2-4] |
| Non-invasive ventilation | 5.523 (47.2%) | 1.959 (52.0%) | 3.564 (45.0%) |
| Median days [Q1-Q3] | 3 [2-6] | 4 [2-8] | 3 [2-5] |
| Invasive ventilation | 3.229 (27.6%) | 1.152 (30.6%) | 2.077 (26.2%) |
| Median days [Q1-Q3] | 4 [2-8] | 5 [2-9] | 3 [2-7] |
| **Length of stay in days (median [Q1-Q3])** |  |  |  |
| In the ICU | 4 [2-7] | 4 [2-7] | 4 [2-7] |
| In the IMCU | 0 [0-2] | 4 [2-8] | 0 [0-0] |
| In the critical care | 5 [3-10] | 9 [5-15] | 4 [2-7] |
| In hospital | 16 [10-25] | 19 [12-29] | 15 [9-23] |
| **Mortality (n. %)** |  |  |  |
| In hospital | 1,118 (9.6%) | 253 (6.7%) | 865 (10.9%) |
